# Compositional Complexity in Text and Images

**DOI:** 10.1162/NOL.a.271

**Published:** 2026-07-09

**Authors:** Helena Balabin, Antonietta Gabriella Liuzzi, Kevin Statz, Marie-Francine Moens, Patrick Dupont, Rik Vandenberghe

**Affiliations:** Laboratory for Cognitive Neurology, Department of Neurosciences, Leuven Brain Institute, KU Leuven, Leuven, Belgium; Language Intelligence and Information Retrieval Lab, Department of Computer Science, KU Leuven, Leuven, Belgium

**Keywords:** compositionality, functional magnetic resonance imaging, multivariate pattern analysis

## Abstract

Compositionality enables us to derive the meaning of a complex whole from the syntax and semantics of its individual parts. While compositional processing has primarily been studied in language, similar principles apply to visual scenes, in which meaning can be derived from their individual parts and the relationships between them. In this study, we therefore aimed to explore whether there is a shared neural basis for compositional processing in text and images. Based on abstract meaning representation graphs for text, commonly used to capture “who is doing what to whom”, and action graph annotations for visual scenes, we defined an analogous graph depth-based notion of compositional complexity. By conducting a functional magnetic resonance imaging experiment in which participants view text-image pairs from the Common Objects in Context-Actions data set, we aimed to identify brain activity patterns related to compositional processing through a combination of univariate and multivariate pattern analysis. Specifically, we hypothesized that there are shared brain regions—such as the pars opercularis, pars triangularis or anterior temporal lobe—involved in processing compositional complexity across text and images. Alternatively, there might exist distinct regions for processing compositional complexity in text and images, without any substantial cross-modal overlap. Our results provided no evidence in support of either hypothesis: no significant neural responses to compositional complexity were observed, in either a shared or modality-specific manner. Given the rigorous control of confounds in our study design, the operationalization of compositional complexity itself may underlie the null result.

## INTRODUCTION

### Compositionality

Compositionality refers to the ability to understand the meaning of a complex whole expression based on the semantics and structure of its constituent parts (Pelletier, 1994; Szabó, 2022). In the human brain, compositional processing is critical for language comprehension, where words are combined to form sentences with distinct meanings (Friederici, 2011). For instance, consider the difference in the following two phrases: “The dog chased the frisbee that the man saw”, and “The dog saw the man chase the frisbee”. Even though the two phrases share a similar number and set of individual words, they differ from each other in their composition and hence in their overall meaning. More specifically, the second phrase introduces a more nested hierarchy (dog → man → frisbee) compared to the first one (dog → frisbee ← man). Similarly, compositional processing is also crucial in human visual perception, where scenes are made up of smaller parts that contribute to the overall perceptual experience (Hafri & Firestone, 2021). Thus, the aforementioned example phrases can be interpreted analogously in a visual setting. Consequently, the ability to process compositional information may not be inherent to a given input modality, but rather reflect a more general modality-independent mechanism (Lambon Ralph et al., 2017).

### Related Work

Compositional processing has primarily been studied in single-modality settings, often in the form of isolated phrases. For instance, much of the existing work on the neural mechanisms underlying compositional processing in language paid particular attention to rigorously controlled two-word phrases (Bemis & Pylkkänen, 2011; Fyshe et al., 2019; Honari-Jahromi et al., 2021). In this context, a comprehensive review by Pylkkänen (2019) has identified a broad combinatory network spanning the angular gyrus, left posterior temporal lobe, left anterior temporal lobe (LATL), left inferior frontal gyrus (LIFG) (also see Kuperberg et al., 2003) and ventromedial prefrontal cortex. In the same vein, Uddén et al. (2022) highlighted the role of the LIFG and LATL as well as the left posterior middle temporal gyrus in compositional processing at the level of sentences, linking their involvement to complexity measures derived from dependency parse trees. This is consistent with findings by Giglio et al. (2024), who demonstrated a connection between grammatical structure and increased connectivity across the pars opercularis of the LIFG and LATL during speaking and listening tasks.

In the visual domain, the notion of compositional scene processing remains less clearly established, reflecting the conceptual asymmetry in how meaning is composed from linguistic versus visual inputs. A review by Epstein and Baker (2019) has identified the parahippocampal place area (PPA) (Epstein & Kanwisher, 1998), retrosplenial complex/medial place area, and occipital place area (OPA) as the main scene-selective cortical regions, building on earlier work on semantic and stuctural relations in scenes (Biederman et al., 1982). However, these regions have primarily been studied in the context of general scene-related rather than compositional processing. These differences among language and vision reflect the specific representational forms of each modality: While semantics provide a consistent basis across linguistic and visual domains, the structural properties that enable composition are not necessarily shared across the two. In another complementary review, Bartnik and Groen (2023) offer three perspectives on scene perception: (a) object-centered (Bar, 2004), (b) space-centered, and (c) affordance-centered. Specifically in the affordance-centered approach (Gibson, 1977), the perception of a scene is primarily defined in terms of the actions enabled by its objects and structure (also see Greene et al., 2016). Furthermore, scene grammars (Võ, 2021; Võ et al., 2019) emerged as a more formalized framework for quantifying how smaller components (i.e., objects or regions) of a scene are compositionally organized into larger coherent structures.

More recently, functional magnetic resonance imaging (fMRI), electroencephalogram or magnetoencephalography experiments increasingly include full sentences, naturalistic images, as well as entire stories and movies (Bhattasali et al., 2020; Chang et al., 2019; Li et al., 2022). Such naturalistic stimuli, often extracted from publicly available image databases frequently used in Machine Learning such as the Common Objects in Context (COCO) data set (Chen et al., 2015; Lin et al., 2014), have enabled the collection of large-scale imaging data sets. As a result, initiatives such as the Natural Scenes Dataset (NSD) (Allen et al., 2022) provide the basis for improved neural decoding analyses at the level of single subjects (Naselaris et al., 2011). Nonetheless, such data sets were not specifically designed for studying compositional processing across modalities. Many are restricted to a single modality (such as the COCO images used in the NSD), while others may not allow for a data-driven parameterization of compositional properties needed for a systematic investigation or do not include repeated stimuli required for reliable neural activity measurements. In conclusion, the precise role of compositionality in processing naturalistic stimuli and potential similarities or differences in processing image or text composition remains unclear to date.

### Rationale

As established previously, compositional properties are often examined for a single modality and in the context of highly controlled conceptual combinations (e.g., adjective noun combinations such as *red boat*) (Frankland & Greene, 2020). Conversely, this study set out to particularly focus on semantic compositional complexity beyond single isolated concepts in a single modality, namely at the level of naturalistic sentences and scenes. Especially in such naturalistic stimuli, the abstract nature of compositional properties makes it inherently challenging to test them empirically. Inspired by Hupkes et al. (2020), who defined five testable compositional properties, namely (a) systematicity, (b) substitutivity, (c) localism, (d) overgeneralization, and (e) productivity, we aimed to measure compositional complexity in a quantifiable manner. In this study, we focused on a representation-based view of compositionality, centered around the semantic and structural properties of a given compositional expression, rather than the cognitive demand required in the production of such an expression. We argue that compositional representations are best captured in graphs, which is why we aimed to leverage graph-based parameters to characterize the compositional complexity of a given naturalistic stimulus. In particular, graph-based measures derived from text or images allowed for an analogous notion of compositional complexity, motivating us to employ paired text-image stimuli to examine the possibly shared neural representations of compositional processing across modalities. Further, this analogous yet independent characterization of compositional complexity in text and images ensured that the localization of potentially shared neural representations remains unbiased. Importantly, in the context of this study, we viewed compositionality as the combination of semantic elements (i.e., nodes of a graph) and their structured relationships (i.e., edges of a graph), abstracting away from modality-specific surface-level syntax. All in all, through systematic manipulation of the graph-based characterizations of image and text stimuli, we aimed to gain further insights into the neural correlates of image- and text-based compositional processing in the brain, and determine whether these mechanisms are shared across modalities.

### The Current Study

In the present study, we aimed to investigate the neural correlates of compositional processing across text and images. To operationalize this aim, we developed a data-driven strategy to automatically select stimuli with examples of varying compositional complexity. Our data-driven strategy required a data set that (a) consists of naturalistic text-image pairs, (b) contains image-based graph annotations that capture essential interactions, and (c) is sufficiently large. Among available data sets, we therefore chose the COCO-Actions (COCO-A) data set (Ronchi & Perona, 2015)—a subset of the COCO data set focusing on human-human or human-object interactions—as a basis for our stimuli selection. We applied both a univariate approach and multivariate pattern analysis (MVPA) to paired text-image stimuli chosen from the COCO-A data set, thereby extending previous work on naturalistic COCO-based stimuli (Allen et al., 2022) to a multimodal setting. Based on abstract meaning representation (AMR) graphs for text, commonly used to capture “who is doing what to whom” abstracted from purely grammatical structure (Knight et al., 2020), and action graph annotations for images, we defined an analogous notion of compositional complexity in each text-image pair. Through binary support vector machine (SVM)-based classification of this compositional complexity measure (high versus low) from fMRI activation patterns within and across images and text stimuli, we aimed to identify regions that exhibit above chance level decoding accuracy to indicate potentially shared or modality-specific neural mechanisms of compositional processing. Specifically, we tested the following main and alternative hypotheses:

1. **Main hypothesis—Modality-independent compositional processing:** There is a shared neural basis for processing compositional complexity across text and image modalities, reflected in shared regions in the univariate analysis or above chance MVPA decoding performance for low versus high compositional complexity for classifiers trained on one modality and tested on the other. Concretely, we hypothesized the involvement of the pars opercularis (see a review by Jeon (2014) highlighting its role in multimodal hierarchical processing), pars triangularis (Pallier et al., 2011), and/or ATL (Lambon Ralph et al., 2017).
2. **Alternative hypothesis—Modality-specific compositional processing:** Distinct brain regions are involved in processing compositional complexity within each modality (i.e., text or images). For example, areas such as the LIFG and LATL may emerge from univariate modality-specific contrast maps or exhibit above chance MVPA decoding accuracy for discriminating high and low compositional complexity in text, and the PPA and OPA for images, but there might not exist any substantial clusters of brain patterns in a cross-modal setting.

## MATERIALS AND METHODS

### Ethics

This study was approved by the ethics committee at the university hospital of Leuven (S68416), subjects were asked to sign a written informed consent form (ICF) in accordance with the Declaration of Helsinki.

### Participants

Eight subjects participated in this fMRI experiment, in accordance with the recent deep data paradigm (Gratton & Braga, 2021; Tuckute et al., 2024), which prioritizes the collection of more stimuli per subject over fewer stimuli in a larger number of subjects. All subjects were required to be Dutch native speakers (i.e., the language spoken at the experimental site). We aimed for a balanced gender and age representation across subjects during recruitment. To be eligible for study participation, a potential subject must meet the following inclusion criteria:

- Right-handedness according to the Edinburgh Handedness Inventory
- Age between 18 and 35
- Normal vision or corrected with contact lenses
- Sufficiently fluency in English (Common European Framework of Reference for Languages (CEFR) level B1 or higher)

To test for English fluency, all potential participants were asked to conduct the Education First Standard English Test (EFSET) to confirm their English proficiency upon filling out the eligibility criteria list (i.e., before the fMRI experiment). The test took around 15 minutes and the result of the test was a proficiency assessment that is in line with the CEFR levels (between A1 and C2). In addition to the CEFR level, we also noted the years of English (L2) acquisition for each participant. Our choice of English (text) stimuli was motivated by our data-driven characterization of compositional complexity in text, which relies on AMR graphs (see Textual Compositional Complexity) that were developed for English rather than for cross-lingual applications (Banarescu et al., 2013). To further minimize linguistic inter-subject variability, we only included Dutch native speakers with sufficient English proficiency in our study. The exclusion criteria are:

- Color-blindness
- History of neurological and/or neuropsychiatric disorders
- History of language development disorder
- The subject takes drugs that can influence their cognitive status
- Implants (excluding braces, teeth fillings, metal wires on the teeth)
- The subject has undergone brain surgery, blood vessel surgery or transplant
- Tattoos or permanent make-up
- Metal objects in the body or having worked in professions with a high risk of metal fragment exposure (e.g., welding)
- Pregnancy
- Other contraindications to MRI based on the local scanning site safety questionnaire

All contraindications were checked using the eligibility criteria list (medical questionnaire) that was given to participants after initial contact together with the ICF. Moreover, if subjects moved excessively during scanning (see Image Preprocessing), we discarded their data, and recruited additional subjects until we reached our targeted sample size.

### Stimuli

As outlined in the introduction, a suitable candidate stimulus set should meet the following criteria:

1. It should consist of text-image pairs describing a naturalistic setting to allow for a joint analysis of image and text
2. Images should contain high-quality (scene) graph annotations, which allow for a robust graph-based characterization of image-based compositional complexity
3. The overall data set should be sufficiently large to allow for filtering examples based on several control variables

Applying the first criterion led to the exclusion of candidate stimulus sets that focus on visual question answering (such as GQA, Hudson & Manning, 2019), often in artificial settings (e.g., the Compositional Language and Elementary Visual Reasoning (CLEVR) data set, Johnson et al., 2017), rather than images with accompanying textual descriptions. Other well-known image-text data sets such as Winoground (Thrush et al., 2022) lacked scene graph annotations, or had annotations of insufficient quality, such as the Visual Genome (Krishna et al., 2017). Especially in the Visual Genome, scene graph annotations do not tend to be constrained to any specific object or relationship types, and the focus lies in complete rather than concise annotations (see the crowdsourcing strategies outlined in Krishna et al., 2017). As a result, many static interaction-free images include up to hundreds of object and relationship annotations, which proved to be counterintuitive for a graph-based characterization of compositional complexity in images. Consequently, we chose the COCO-A data set (Ronchi & Perona, 2015), because it is inherently constrained to human-human or human-object interactions based on a fixed set of visual action verbs. Specifically, we used the COCO-A version with an annotator agreement of at least three persons, yielding 4,413 unique images with at least five captions each (mean number of captions = 7.00 ± 2.45).

#### Textual compositional complexity

There are various approaches for characterizing the composition of text, such as dependency parsing, constituency parsing, or semantic role labeling (Jurafsky & Martin, 2024). As mentioned in the introduction, we aimed to create an analogous notion of compositional complexity in text and images by focusing on semantic representations and relationships among them. To achieve this goal, we adopted AMR, a semantic formalism, which encodes the meaning of a sentence as a rooted, directed, acyclic graph (Flanigan et al., 2014). More specifically, each node in the AMR graph denotes a concept (e.g., a human or an object), whereas edges represent semantic relations among the concepts (e.g., a frame argument structure representing a “who is doing what to whom” relation between three concepts). Contrary to dependency or constituency parsing, AMR graphs therefore offer more semantically grounded representations, which are explicitly designed to abstract away from variations in surface-level syntax caused by variations in parts-of-speech that convey the same meaning. In the context of our study, AMR graphs offered two main advantages. First, since AMR graphs directly use concepts and their relationships as nodes and edges (Banarescu et al., 2013), without the overhead of prepositions and determiners found in dependency trees, they could be meaningfully compared to action graphs derived from COCO-A images. Secondly, as a result of using concepts rather than all possible words as nodes, slightly differently phrased sentences with identical meaning (e.g., different word orders) were represented by the same AMR graph, making them more robust to small variations in wording. For instance, the phrases “he described her as a genius” and “his description of her: genius” would both be represented by the same AMR graph, in which “describe” would be the root node, with edges to the “he”, “she”, and “genius” leaf nodes (Banarescu et al., 2013).

Consequently, to derive the compositional complexity in text, we generated an AMR graph for each COCO-A caption using the AMRBart model (Bai et al., 2022), which has demonstrated strong performances on several AMR benchmark tasks (Groschwitz et al., 2023). Then, to differentiate between captions of varying compositional complexity based on their AMR graphs, we parameterized complexity in terms of graph depth, with a higher depth indicating a more complex caption. Note that unlike in the case of dependency parsing, AMR graphs are not necessarily trees; hence we defined depth as the longest of the shortest (directed) paths between any two nodes in the graph. Alternatively, we parameterized complexity in terms of the total number of edges, with an edge count above the median edge count across all stimuli indicating a more complex caption, and an edge count below the median indicating a less complex caption. Further, we excluded all attributes (i.e., additional relationships between a concept node and a constant, such as a number) from the AMR graphs to solely base our measures on edges between concepts.

#### Visual compositional complexity

Compared to the existing approaches for text, the characterization of visual compositional complexity in naturalistic scenes is generally less grounded in well-established frameworks. Most characterizations target spatial relationships between objects in an image (e.g., scene grammars, Võ, 2021). Here, we chose to focus on relationships among humans or between humans and animals or objects, as defined by the annotation constraints of the COCO-A data set, for three key reasons. First, such relationships facilitated a robust graph-based characterization, given that both nodes (i.e., humans or animals/objects that they interact with) and edges (i.e., visual actions derived from a fixed list of verbs, see Ronchi & Perona, 2015) in such COCO-A graphs are clearly identifiable and salient in a given scene. These structured relationships closely mirror text-based AMR graphs, which are also based on a fixed set of semantic relationships (Banarescu et al., 2013), thereby enabling a more direct comparison between the two modalities. Additionally, such relationships directly convey possibilities for action, and are thus in line with the affordance-centered approach (Greene et al., 2016; Greene & Oliva, 2009; Hafri et al., 2018) of scene perception. Second, emphasizing human-centered interactions aligned the visual compositional complexity more closely with the textual captions, which primarily describe the actions of the main subjects. Like AMR graphs, COCO-A graphs abstract away from variations in surface-level characteristics (e.g., background clutter) to focus on who is doing what to whom, offering a comparable notion of semantic compositionality between text- and image-based graphs. Third, this constraint enabled us to better isolate the specific effect of the graph depth and number of edges on the resulting fMRI representations. Analogous to the text, we parameterized the compositional complexity of the corresponding images based on the graph depth (i.e., the longest of the shortest (directed) paths between any two nodes), or alternatively, the total edge count.

#### Control variables

To address potentially correlated covariates to our chosen compositional complexity measures, the following variables were controlled for when selecting the stimuli:

- **Text**
  – **Caption length:** Captions should contain between eight and twelve words. The specific range was chosen based on maximizing the remaining number of candidate stimuli given the length distribution of the captions in the COCO-A data set (10.66 ± 2.60 words on average).
  – **Presence of verbs:** Captions should contain at least one verb.
  – **Number of nodes (AMR):** To avoid outliers, we excluded captions whose AMR graphs contained an unusually low or high number of nodes. Specifically, we only included captions with a number of nodes between the 5% and 95% quantile across all graphs (i.e., between four and seven nodes).
- **Images**
  – **General image density:** To ensure that our graph-based characterization of compositional complexity in images is not confounded by other factors, such as image saturation, background, or general detail and variety, we controlled for these general image characteristics. Leveraging the IC9600 model (Feng et al., 2023), we predicted an image density score (ranging from 0 to 1) for each COCO-A example and selected images with scores between 0.2 and 0.8 (mean image density score = 0.52 ± 0.11).
  – **Aspect ratio:** Images should have an aspect ratio (i.e., number of width pixels divided by the number of height pixels) of at least 1.0 and at most 2.0, i.e., images should either be square or horizontal.
  – **Image quality:** Images must be in color and have a minimum height of 300 pixels.
  – **Number of people:** Using the image segmentation metadata of the original COCO data set for every example included in COCO-A enabled us to control for the number of people in each image. More specifically, we included images using a range of least two and at most 11 persons, chosen based on distribution of the number of people in the COCO-A data set (5.13 ± 4.13 persons on average).
  – **Number of nodes (COCO-A):** Once again, we controlled for the number of nodes in the COCO-A graphs, excluding images with an unusually low or high number of nodes using the [0.05, 0.95] quantile range across all graphs (i.e., between four and 12 nodes).

#### Stimulus selection

**Figure 1.**
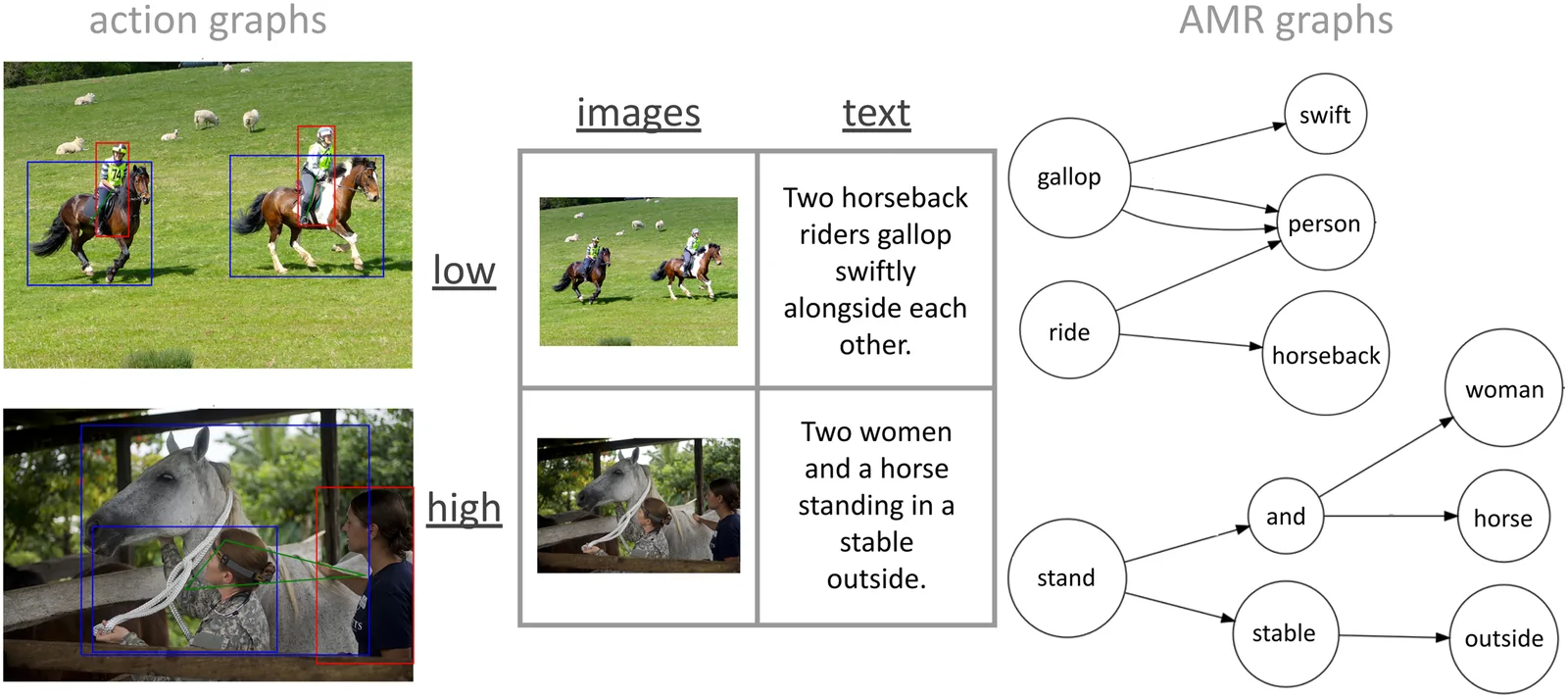
Example text-image pairs with low and high compositional complexity, respectively. The text-image pairs, selected from the COCO-A data set, were chosen based on the graph-based complexity measures of their corresponding action graphs (left) and abstract meaning representation (AMR) graphs (right). During the experiment (gray box), subjects only saw the raw images or text without any graph overlays.

Taking our control variables and parameters together, we applied a systematic stimulus selection procedure. Initially, we filtered the COCO-A data set by applying our predefined control variables, yielding 957 unique images and 5,600 total image-text pairs. For the filtering process, we first considered all possible image-text pairs as separate examples, sequentially applying text-based and image-based filtering steps. From this refined subset, our target was to select a set of *n* = 252 stimuli, stratified equally between two compositional complexity levels: pairs with matching textual and visual graph depths of (1, 1) and (2, 2). The choice of this binary depth characterization, rather than a parametric one, was motivated by the need to ensure comparability across image and text modalities and to provide a sufficient number of examples at each depth level for reliable analyses (see Table 1 for an overview of the depth level pairings for the 5,600 text-image pairs). To guarantee that the aforementioned control variables are balanced across the two resulting complexity levels, we performed nearest neighbor-based propensity score matching using PsmPy (Kline & Luo, 2022) to pair high- and low-complexity stimuli with similar covariate characteristics. During this matching process, we addressed potential duplications by retaining only one example per unique image when multiple captions existed with identical textual complexity levels. To ensure robust selection, we initially over-sampled by applying a 30% buffer, resulting in an initial set of 328 stimuli (i.e., 164 matched pairs of low and high complexity stimuli). We then applied two final manual quality control steps. First, we removed all text-image pairs containing images with overly prominent text (e.g., images of people holding signs with text). Second, we eliminated pairs where humans were not the primary subject of the caption (e.g., *“A bus that is sitting in the street”*). From the remaining curated set of text-image pairs, we randomly selected the final *n* = 252 stimuli for our experiment. The values of all control variables in the final stimulus set are summarized in Table 2, and two example text-image pairs with low and high compositional complexity, respectively, are shown in Figure 1.

**Table 1.** Characterization of the initial 5,600 image-text pairs in the COCO-A data set, filtered for the previously described control variables. Each cell denotes the number of image-text pairs that have an image-based COCO-A graph depth listed in its row header, and a text-based abstract meaning representation (AMR) graph depth listed in its column header, respectively. The underlined cells form the basis for pairs with matching textual and visual graph depths of either (1, 1) or (2, 2). Note that this table describes the number of stimuli prior to the de-duplication of images with multiple captions.

**AMR/COCO-A**	**1**	**2**	**3**	**4**	**5**
**1**	559	1399	562	72	3
**2**	371	1505	469	45	8
**3**	87	315	137	11	0
**4**	8	29	18	2	0

**Table 2.** Mean (± standard deviation) values of the control variables for high (image- and text-based depth values of (2, 2)) and low compositional complexity stimuli (depth characterization of (1, 1)) of the final stimulus set of *n* = 252 text-image pairs. While the number of nodes (AMR) were derived from the captions, the number of nodes (COCO-A) were derived from the image-based action graphs. Significant differences based on a two-sided *t*-test (*p* < 0.05) are indicated with an asterisk.

**Control Variable**	**High Complexity**	**Low Complexity**	***p*-value**
**Caption length**	9.433 ± 1.312	9.598 ± 1.218	0.241
**Number of nodes (AMR)**	5.329 ± 1.042	5.427 ± 1.018	0.394
**Number of people**	4.427 ± 2.676	4.555 ± 2.649	0.664
**General image density**	0.517 ± 0.074	0.528 ± 0.086	0.202
**Aspect ratio**	1.427 ± 0.118	1.394 ± 0.163	0.037*
**Image quality (height pixels)**	440.945 ± 67.279	453.145 ± 61.218	0.059
**Number of nodes (COCO-A)**	7.287 ± 2.439	7.085 ± 2.777	0.488

### Experimental Procedure

Overall, our experimental paradigm followed an event-related design, in which we showed text and images separately, displaying images without explicit graph structure overlays. Each of the 252 text–image pairs was split across participants such that each participant saw only one modality (either text or image) per pair. Across all participants, each item was shown in both modalities, with half of the participants viewing the text and the other half viewing the corresponding image. As a result, all participants were exposed to the full set, but in a single modality per stimulus. This guaranteed that no participant ever paired a caption to a previously seen image or vice versa, thus avoiding cross-coding effects. Each stimulus was presented six times, yielding a total of (126 captions + 126 images) × 6 = 1,512 trials per participant. The experiment comprised three sessions consisting of twelve runs with 50 trials each, lasting approximately 5 min. Each run contained two start blank trials, 42 stimuli trials interspersed with four blank trials, and two final blank trials. We pseudo-randomized the trial order while maintaining a balanced distribution of the two experimental factors: modality (image versus text) and compositional complexity (high versus low). Moreover, to avoid memory effects, repetitions of a stimulus were not included in the same run. To further mitigate potential sequential effects, we stratified the modality and complexity distributions in six smaller segments within each run. A pseudo-randomized stimuli order was applied to each segment. Each stimulus was presented for 3 s, with an inter-stimulus interval (ISI) of another 3 s. In addition to the functional runs, the first session also included an anatomical scan. Prior to the functional runs, a brief practice run with a distinct set of practice stimuli preceded the main experiment to familiarize the participants with the procedure. Stimuli were presented using PsychoPy (Peirce et al., 2019). To ensure optimal synchronization between stimulus presentation and scanning, each run was started by a trigger from the MR scanner. Further, to lock the stimulus duration to the frame rate of the 60 Hz monitor used at the MR scanner, we specified the exact number of frames shown in the PsychoPy scripts based on the stimulus duration and frame rate (i.e., 180 frames for an image or a text, with a subsequent ISI of 180 frames).

### Behavioral Measures

To verify that participants were actively processing the presented stimuli, we included a yes/no recognition task after each session. In more detail, this behavioral task included 50 of the presented stimuli (25 texts and 25 images) and 50 lure stimuli (another 25 experimental texts and 25 images) that were randomly chosen from the COCO-A data set and were not shown during the experiment. Each session contained a different set of lure stimuli. Participants were asked to indicate whether they have seen the candidate stimulus during the experiment or not, without an explicit time limit. For each participant, we report the recognition accuracy averaged across the three sessions.

### Image Acquisition

Functional and structural images were collected using a Philips Achieva dstream 3T scanner equipped with a 32-channel head volume coil. For the functional images, we used T2* echoplanar images comprising 48 transverse slices (repetition time: 1.5 s, echo time: 30 ms, voxel size: 2 × 2 × 3.3 mm^3^, sensitivity encoding, SENSE, factor: 2), covering the entire brain (field of view, FOV: 224 × 224 × 158 mm^3^). Moreover, each run was preceded by four dummy scans, allowing for a steady state magnetization of the blood oxygenation level dependent (BOLD) imaging acquisition. For structural images, we employed a sequence consisting of a T1-weighted 3D turbo-field-echo sequence (repetition time: 5.43 ms, echo time: 2.41 ms, and 1 mm isotropic resolution). All imaging data was converted to Brain Imaging Data Structure (BIDS) format (Gorgolewski et al., 2017).

### Image Preprocessing

Before preprocessing, we first performed an initial quality control step using the visual inspection files offered in the MRIQC framework (Esteban et al., 2017). In particular, we controlled for and discarded runs from subjects with excessive movement, defined as more than 20% of volumes exceeding a frame-wise displacement threshold above 1.0 mm. Data that passed initial quality control was preprocessed with default parameters in fMRIPrep (Esteban et al., 2019), comprising the following steps: (a) Head-motion correction, (b) slice time correction, (c) co-registration of functional and anatomical scans, and (d) normalization to the Montreal Neurological Institute (MNI) space (using the MNI152NLin2009cAsym template), and (e) spatial smoothing with a Gaussian kernel of 6 mm full-width at half maximum. Next, we estimated two types of response estimates (i.e., betas) per voxel from the preprocessed fMRI time series using general linear models (GLMs): (a) Four condition-specific betas (i.e., high/low compositional complexity × image/text) using the Nilearn package in python (Gorgolewski et al., 2011), which modeled the conditions together with a cosine drift model and six motion regressors derived from fMRIprep, as well as (b) trial-wise betas with the GLMsingle (Prince et al., 2021) framework, which is specifically suited for denoising overlapping hemodynamic response functions. For both types of betas, we derived an alternative set for which we performed the same analysis outlined below, using GLMs that included the following confounds as predictors: Caption length, number of nodes in the text-based graph, number of nodes in the image-based graph, image density, image aspect ratio and the number of people in the image (with a value of zero whenever a confound is not applicable, such as the aspect ratio for text). Additionally, GLMsingle enabled us to measure the general variance explained per voxel given the experimental stimuli (regardless of the specific modality or complexity) versus the blank trials, as well as the test–retest reliability of each voxel.

### Statistical Analyses

To test which brain regions are involved in compositional processing in (a) text and images and (b) whether they are shared across modalities, we employed two types of analyses: A univariate analysis and a searchlight-based MVPA (Haxby et al., 2001; Kriegeskorte et al., 2006), both implemented in Nilearn. First, the univariate analysis was based on the condition-specific betas, masked using the subject-specific gray matter mask with a probability threshold of > 0.3. Using this subset of voxels, the following modality-specific contrasts were calculated: Highly versus lowly compositionally complex images, and highly versus lowly compositionally complex texts. In addition, we tested the following: (a) The main effect of compositionality, (b) the main effect of modality, and (c) the interaction effect between compositionality and modality to identify regions where the effect of compositionality differs between images and text. Then, at the group-level, we performed a fixed effect analysis based on a one-sample *t*-test for each contrast (using the following thresholds: uncorrected voxel-level *p* < 0.001 and family-wise error (FWE)-corrected cluster-level *p* < 0.05). Moreover, we calculated the conjunction of the group-level t-maps for highly versus lowly compositionally complex stimuli in each modality to identify possible clusters of shared neural mechanisms involved in processing compositional complexity across the two modalities.

However, since a homogeneous functional localization across participants may not always hold true, we aimed to conduct additional analyses based on functional localization within each participant. More specifically, the aim was to use all runs from the first session to identify participant-specific voxels responsive to compositional complexity of any kind, defined by the contrast of highly versus lowly compositionally complex stimuli across modalities, thresholded at uncorrected voxel-level *p* < 0.001. The resulting participant-specific masks were intended to be used as regions of interest to derive mean activation values from the second and third sessions to evaluate the main effects of compositionality for each modality, as well as their interaction. Finally, we aimed to assess group-level consistency by generating probabilistic maps showing the percentage of participants with significant activation in each voxel, as identified by the functional localizer.

Second, the MVPA consisted of a binary classification task, in which we trained and tested linear SVM classifiers (with default hyperparameters as defined in the scikit-learn python package, Pedregosa et al., 2011), chosen because of their effectiveness in class separation, to distinguish between low and high compositional complexity levels either (a) within or (b) across modalities, using the data from all three sessions. First, for each subject, we restricted the set of voxels at whole-brain level by using those that carried experimentally driven signal (i.e., those that passed the default explained variance threshold *R*^2^ in the ON-OFF model in GLMSingle, determined by a two-component Gaussian Mixture Model, see Prince et al., 2021), and that were sufficiently reliable (i.e., voxels passing a test–retest reliability Pearson correlation threshold of >0.1). Then, for these voxels, we averaged the trial-wise beta estimates across the three repetitions of each stimulus, to subsequently use them as input for the MVPA. Next, we trained and tested the SVM classifiers on the averaged beta estimates for each voxel with a searchlight, using a radius of 6 mm. For the within-modality decoding, we trained and tested using either exclusively text or images in a stratified five-fold cross-validation setting, measuring the accuracy to discriminate between highly vs lowly compositionally complex stimuli for each voxel and test fold. Conversely, for the cross-modal decoding task, we both (a) trained on all available text and tested on all image stimuli and (b) trained on all images and tested on all text stimuli. By including *n* = 252 stimuli per modality per subject, we aimed to obtain more robust decoding accuracies. The resulting outcomes were the subject-specific accuracy maps averaged across the five test folds, together with the respective standard deviation maps.

Then, we again conducted a fixed effect analysis, in which we applied a one-sided *t*-test to identify clusters in which the classification accuracy differed significantly from chance (based on the following thresholds: uncorrected voxel-level *p* < 0.001 and FWE-corrected cluster-level *p* < 0.05). Consequently, we derived three outcome measures: The identification of clusters in the text- and image-based MVPA patterns, and in the cross-modal decoding setting in either direction (i.e., [a] text-to-image or [b] image-to-text). The existence of such clusters in the group-level t-map derived from (a) or (b) would provide evidence for our hypothesis of shared neural mechanisms involved in processing compositional complexity in a modality-independent manner, whereas their absence could be interpreted in favor of the alternative hypothesis focusing on modality-specific processing.

## RESULTS

A total of eight participants completed the study (four female, four male, mean age 24.9 ± 2.4 years, [22–29]). All participants were native Dutch speakers who passed the pre-defined English proficiency threshold (four B1/B2, four C1/C2 according to the EFSET CEFR scale), and had 8.0 ± 2.0 years of L2 English acquisition.

### Behavioral Results

All participants completed a yes/no recognition task, which asked them to distinguish between 50 previously seen stimuli and 50 lures. The mean recognition accuracies, averaged across the three sessions for each subject, are listed in Table 3. For one subject, only two recognition tasks were completed, though scanning data was acquired for all three sessions. Across all subjects, the mean recognition accuracy was 0.844 ± 0.039, ranging from 0.693 ± 0.036 to 0.940 ± 0.054. All individual recognition accuracies were significantly above chance level (*p* < .001, see Table 3). Further, we calculated *d*′ values for each participant, using condition-specific hit rates (modality × complexity) and a false alarm rate derived from lure items. A pairwise Wilcoxon signed-rank tests with Bonferroni correction for multiple comparisons on *d*′ scores, with modality (images or text) and complexity (high or low) as within-subject factors, revealed no significant effect of modality (*W* = 7, *p* = 0.445, *r* = 0.61), complexity (*W* = 16, *p* = 1.000, *r* = 0.11), or their interaction (*W* = 15, *p* = 1.000, *r* = 0.17), indicating that recognition performance was broadly comparable across conditions.

**Table 3.** Recognition accuracy on the yes/no recognition task for each subject, averaged across sessions. In the recognition task, participants were asked to distinguish between 50 lure stimuli (25 images/25 texts) and 50 stimuli that were included in the experiment (25 images/25 texts). Each row indicates the recognition accuracy averaged across the three sessions for a given subject (except for subject 9, who did not complete the recognition task after the third session), together with the respective standard deviation.

**Subject**	**Accuracy (Mean ± Standard Deviation)**
2	0.826 ± 0.005***
3	0.851 ± 0.079***
4	0.855 ± 0.005***
5	0.896 ± 0.055***
6	0.795 ± 0.059***
7	0.940 ± 0.054***
8	0.693 ± 0.036***
9	0.895 ± 0.018***
**Mean**	**0.844 ± 0.039** ***

*** Indicates *p* < .001, based on a comparison against a null hypothesis of random guessing (chance = 0.50).

### Univariate Analysis of Compositional Complexity

**Figure 2.**
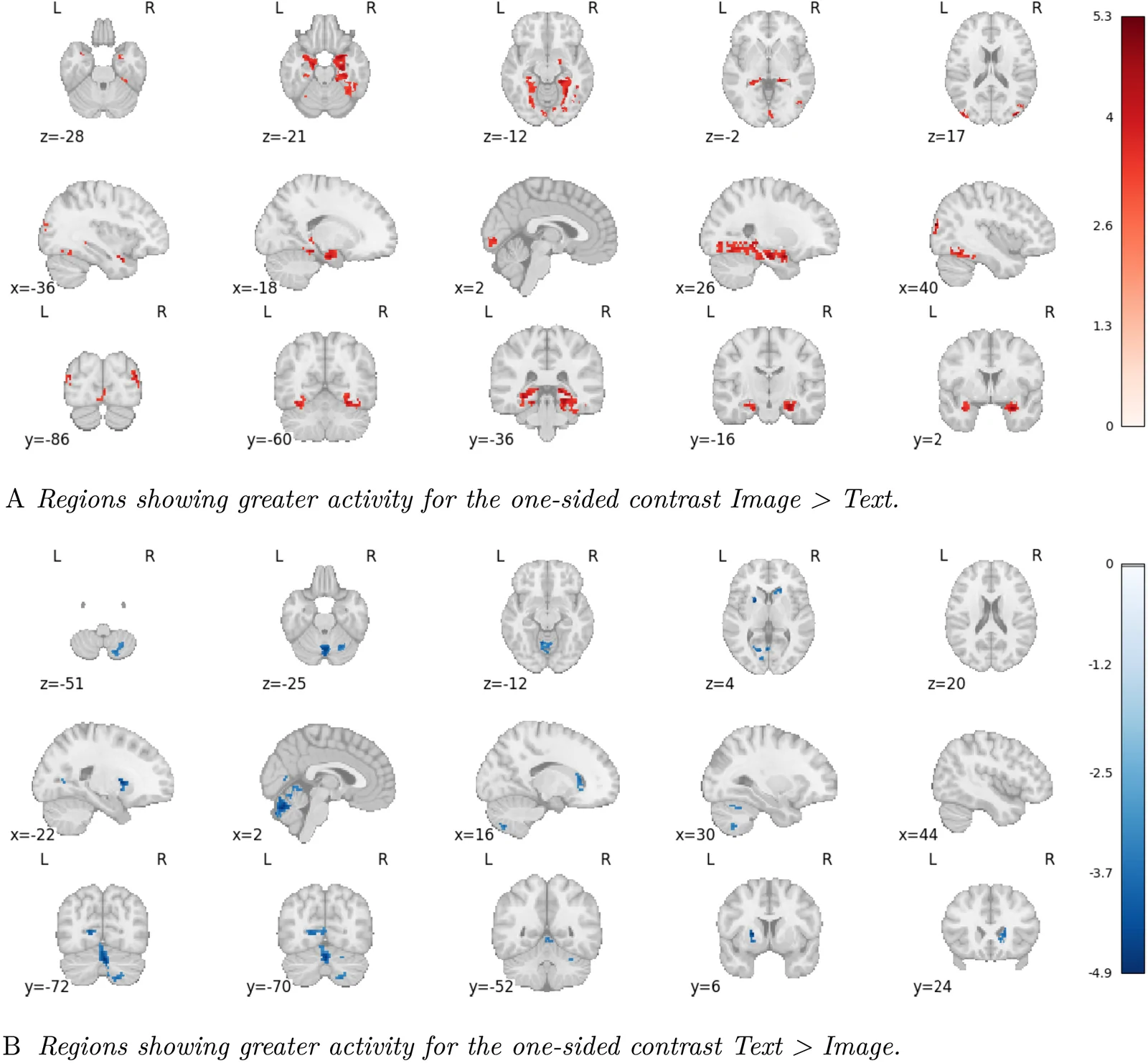
Univariate group-level main effect of modality. L = left, R = right. The statistical thresholds employed are voxel-wise *p* < 0.001 and cluster-level family-wise error correction of *p* < 0.05. Results are displayed on the MNI152 template.

All runs from all eight subjects passed the pre-defined quality control threshold for excessive movement (20% of the volumes exceeding a frame-wise displacement above 1.0 mm, see Image Preprocessing), hence, no data needed to be discarded. At the group level, based on the pre-defined voxel- and cluster-level significance thresholds (see Statistical Analyses), we found no significant main effect of compositionality. Conversely, the main effect of modality yielded significant clusters of activation (see Figure 2). The one-sided *Image - Text* contrast (see Figure 2A) revealed significant clusters primarily located around the ventral visual pathway. Markedly, significant activation for the main modality contrast did not include the primary visual cortex but emerged in more downstream regions of the ventral visual pathway. More specifically, clusters of significant activation are located in the fusiform gyrus, including the fusiform face area (FFA), and the parahippocampal gyrus, which encompasses the PPA. These clusters appeared as discrete rather than contiguous regions of activation along the ventral visual pathway, possibly as a result of thresholding. The reverse one-sided *Text - Image* contrast yielded significant clusters of activation in the cerebellum, anterior putamen, and right caudate nucleus (see Figure 2B, most notably the middle row). No significant interaction between modality and compositionality was found. Similarly, the conjunction analysis yielded no significant results, i.e., no overlap of significant activation was found across regions modulated by compositional complexity within images and text, respectively, yielding no shared neural responses of compositional processing across modalities. Notably, even at the individual subject level (see Figure 7), the compositionality contrast (using combined text and image data) did not yield activation masks of sufficient spatial extent to support the extraction of a robust functional localization mask. Consequently, the functional localization approach was omitted for the remainder of the analyses. Additional analyses including the extra confounds (see Image Preprocessing) led to analogous results and are shown in Supplementary Figure A1 (Supporting Information can be found at https://doi.org/10.1162/NOL.a.271).

For completeness, the results of the first-level analyses for each of the eight individual subjects are presented in Figures 4–8 (at uncorrected voxel-level *p* < 0.001 and no cluster-level correction), showing the main modality, compositionality within images, compositionality within text, main compositionality and interaction contrasts, respectively. While the main modality contrast (see Figure 4) elicited the strongest significant differences between the image and text conditions within subjects as well, the remaining contrasts vary substantially, especially across subjects. This variability does not only consist of differences in the spatial distribution of the clusters, which may partly reflect the applied statistical threshold, but also in the directionality of the response (e.g., the two subjects shown in the upper right quadrant of Figure 7).

### Multivariate Pattern Analysis of Compositional Complexity

**Figure 3.**
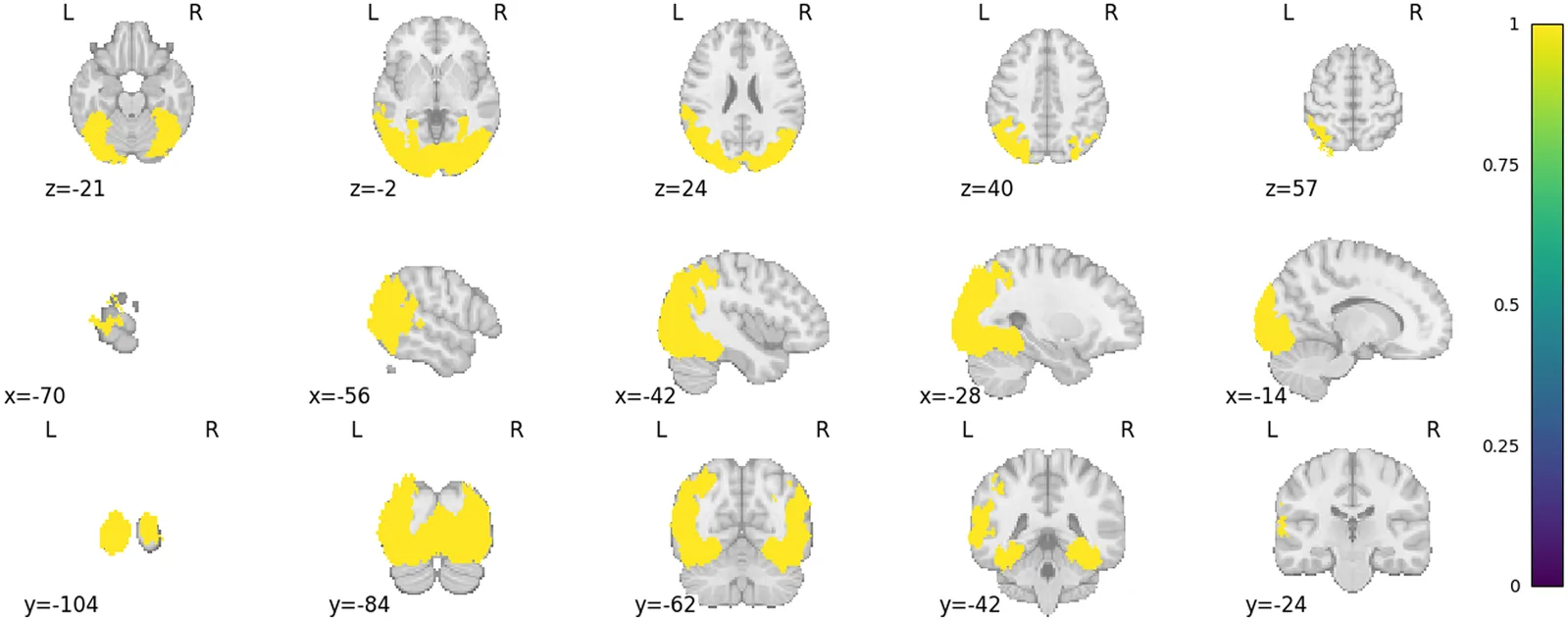
Group-level union of subject-level brain masks based on voxels that pass the default explained variance and test–retest variability threshold. L = left, R = right. Results are displayed on the MNI152 template.

For the decoding analyses, the spatial coverage of the search space was restricted by the individual masks (the combined group mask is shown in Figure 3), which were derived by filtering the total set of voxels to those that exceed the default explained variance threshold in the GLMSingle ON-OFF model and had a test–retest variability of *r* > 0.1. As a result, the MVPA decoding tasks were restricted to the occipital lobe, and to a smaller extent, the parietal and temporal lobes. At the group level, based on the pre-defined significance thresholds (see Statistical Analyses), no clusters of significant neural activation were found for any of the four decoding task configurations: Within-image, within-text, text-to-image and image-to-text. Specifically, the absence of significant neural activation in the cross-modal configurations reveals no shared neural response to compositional processing across modalities in the MVPA decoding setup. Additional results for the MVPA decoding tasks based on single-trial beta estimates that control for the extra confounds (i.e., caption length and the number of nodes in the associated AMR graphs for text stimuli, and general image density, aspect ratio, image quality, number of people and number of nodes in the associated COCO-A graphs for image stimuli) are shown in Supplementary Figure A2.

**Figure 4.**
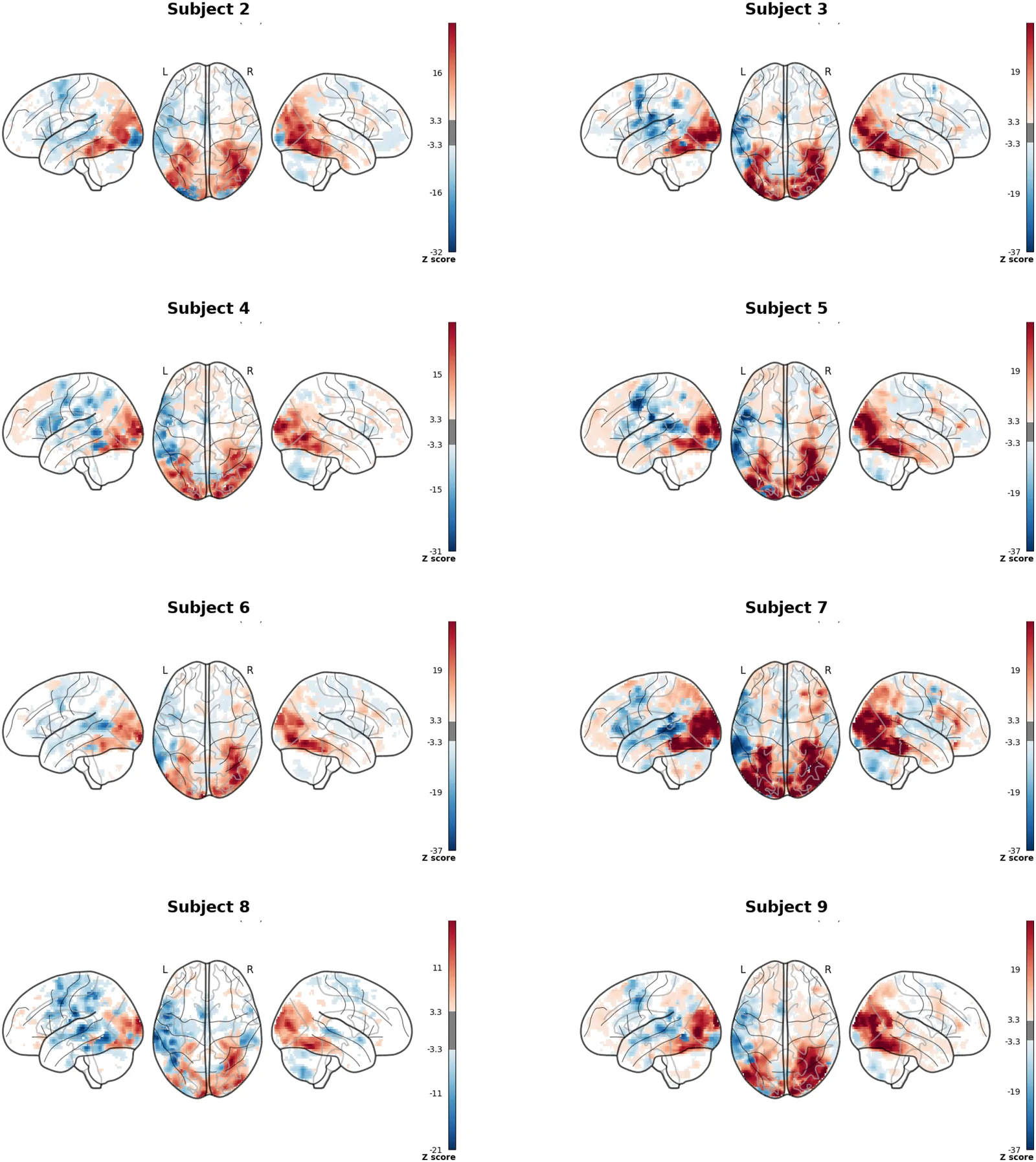
Individual subject-level activation maps for the main modality contrast, visualized on a glass brain in MNI152 space. Each panel represents one subject, with the respective *z*-score map showing the spatial distribution of significant voxels, thresholded at *p* < 0.001 uncorrected with no cluster-level correction, and a minimum peak distance of 8 mm. Positive *z*-scores (shown in red) indicate a stronger response to images, whereas negative *z*-scores (shown in blue) indicate a stronger response to text.

**Figure 5.**
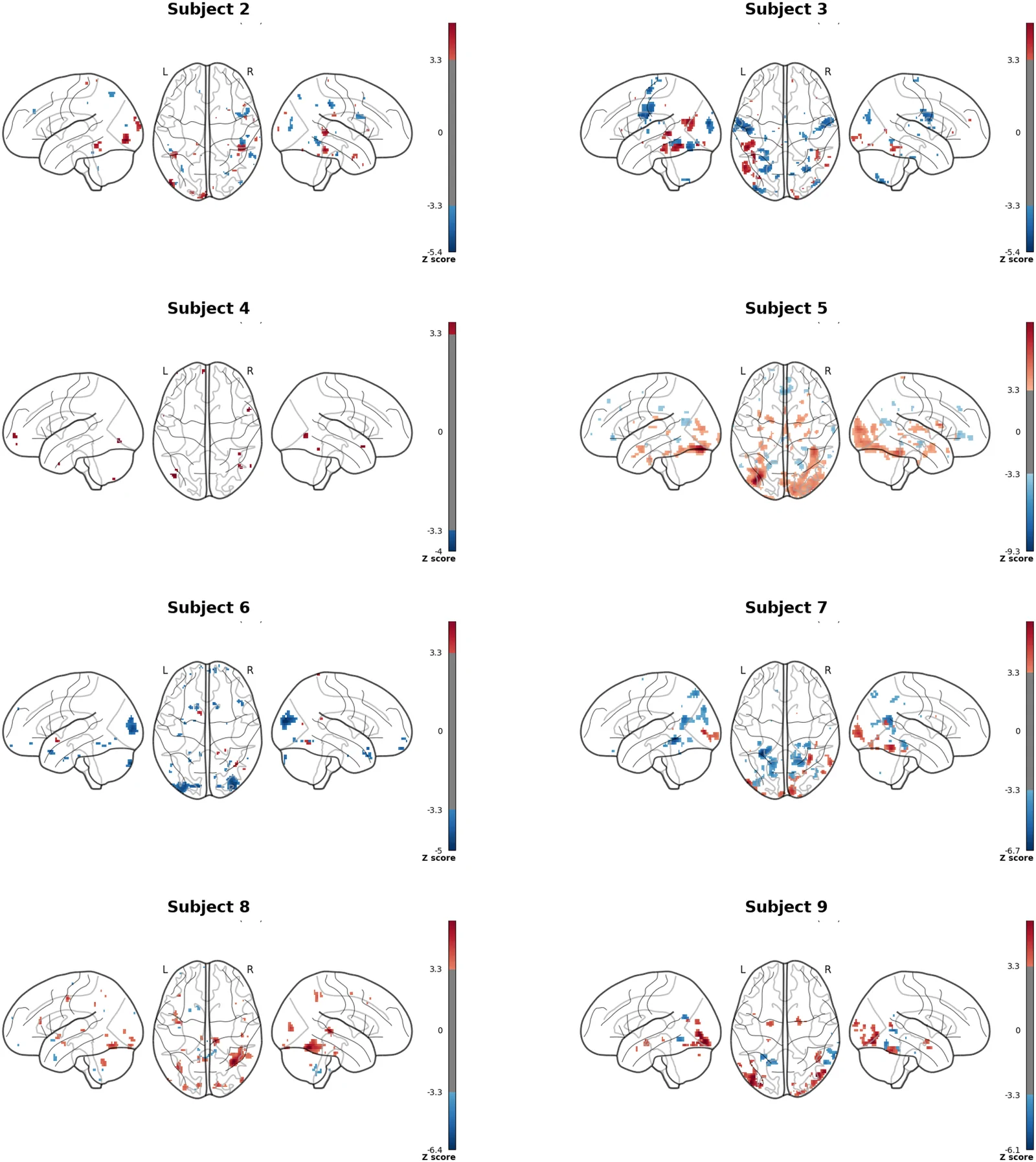
Individual subject-level activation maps for the compositionality contrast within images. Each panel represents one subject, with the respective *z*-score map showing the spatial distribution of significant voxels, thresholded at *p* < 0.001 uncorrected with no cluster-level correction, and a minimum peak distance of 8 mm. Positive *z*-scores (shown in red) indicate a stronger response to highly compositionally complex images, whereas negative *z*-scores (shown in blue) indicate a stronger response to lowly compositionally complex images.

**Figure 6.**
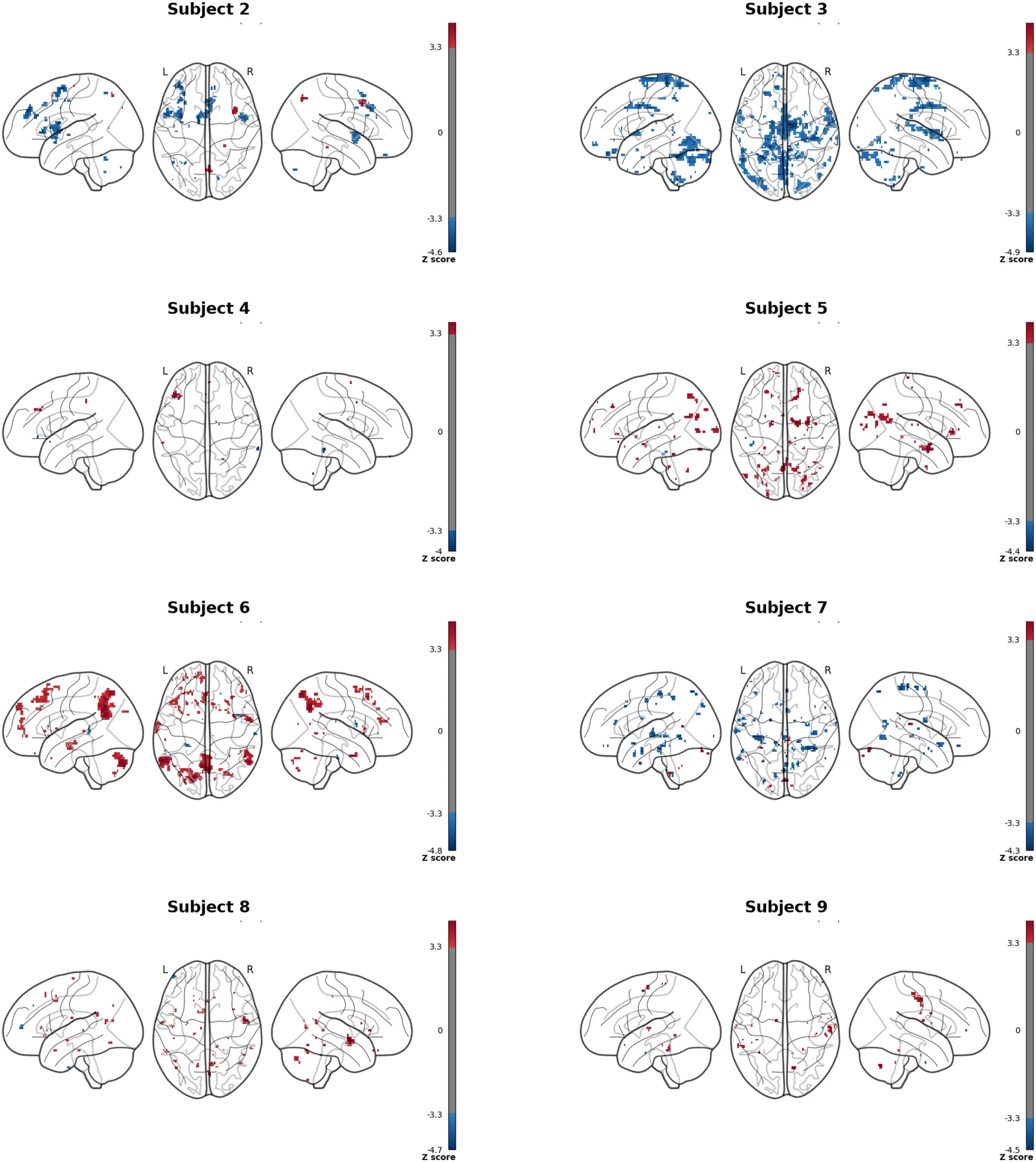
Individual subject-level activation maps for the compositionality contrast within text. Each panel represents one subject, with the respective *z*-score map showing the spatial distribution of significant voxels, thresholded at *p* < 0.001 uncorrected with no cluster-level correction, and a minimum peak distance of 8 mm. Positive *z*-scores (shown in red) indicate a stronger response to highly compositionally complex text, whereas negative *z*-scores (shown in blue) indicate a stronger response to lowly compositionally complex text.

**Figure 7.**
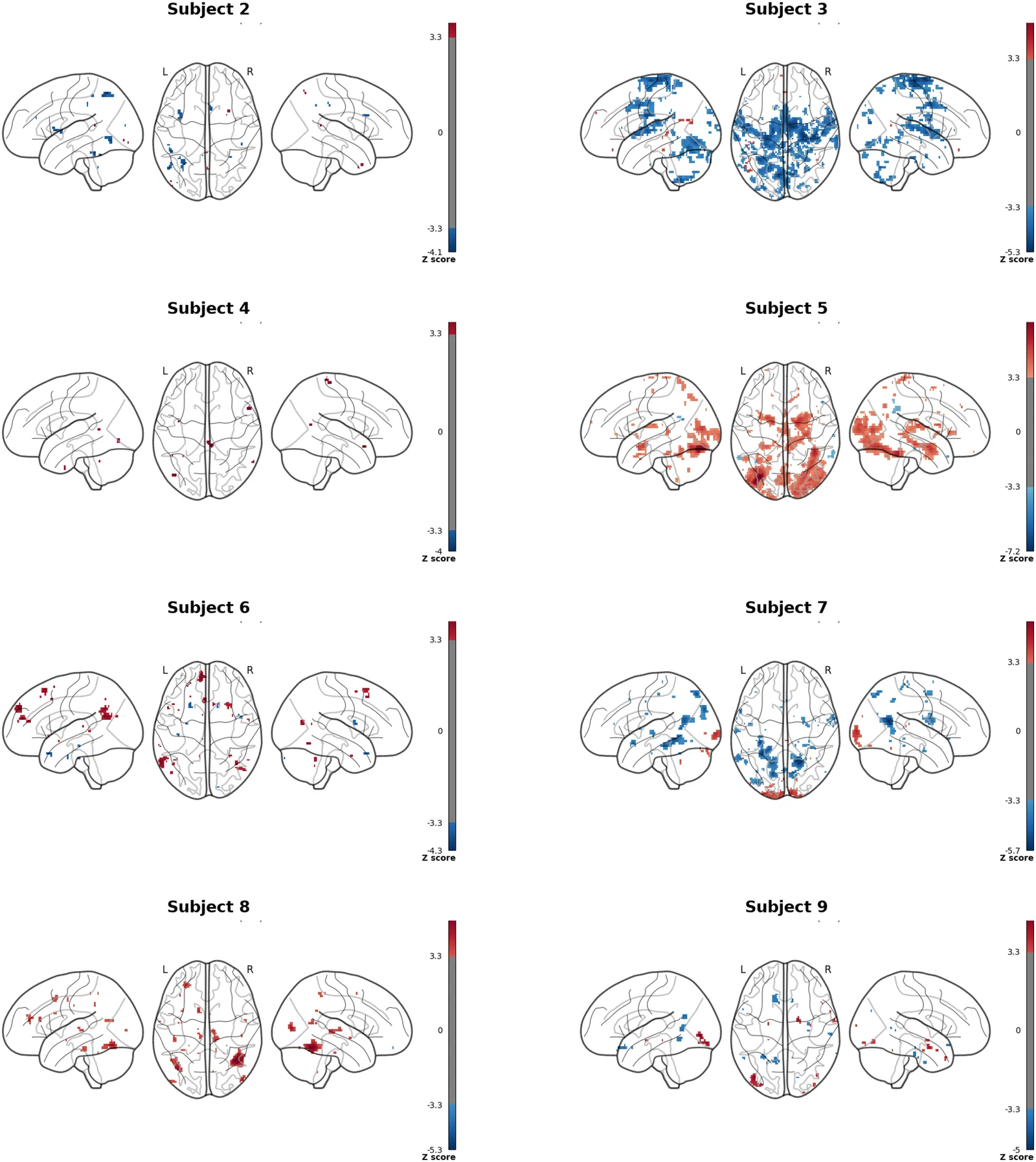
Individual subject-level activation maps for the main compositionality contrast (across text and images combined). Each panel represents one subject, with the respective *z*-score map showing the spatial distribution of significant voxels, thresholded at *p* < 0.001 uncorrected with no cluster-level correction, and a minimum peak distance of 8 mm. Positive *z*-scores (shown in red) indicate a stronger response to highly compositionally complex inputs, whereas negative *z*-scores (shown in blue) indicate a stronger response to lowly compositionally complex inputs.

**Figure 8.**
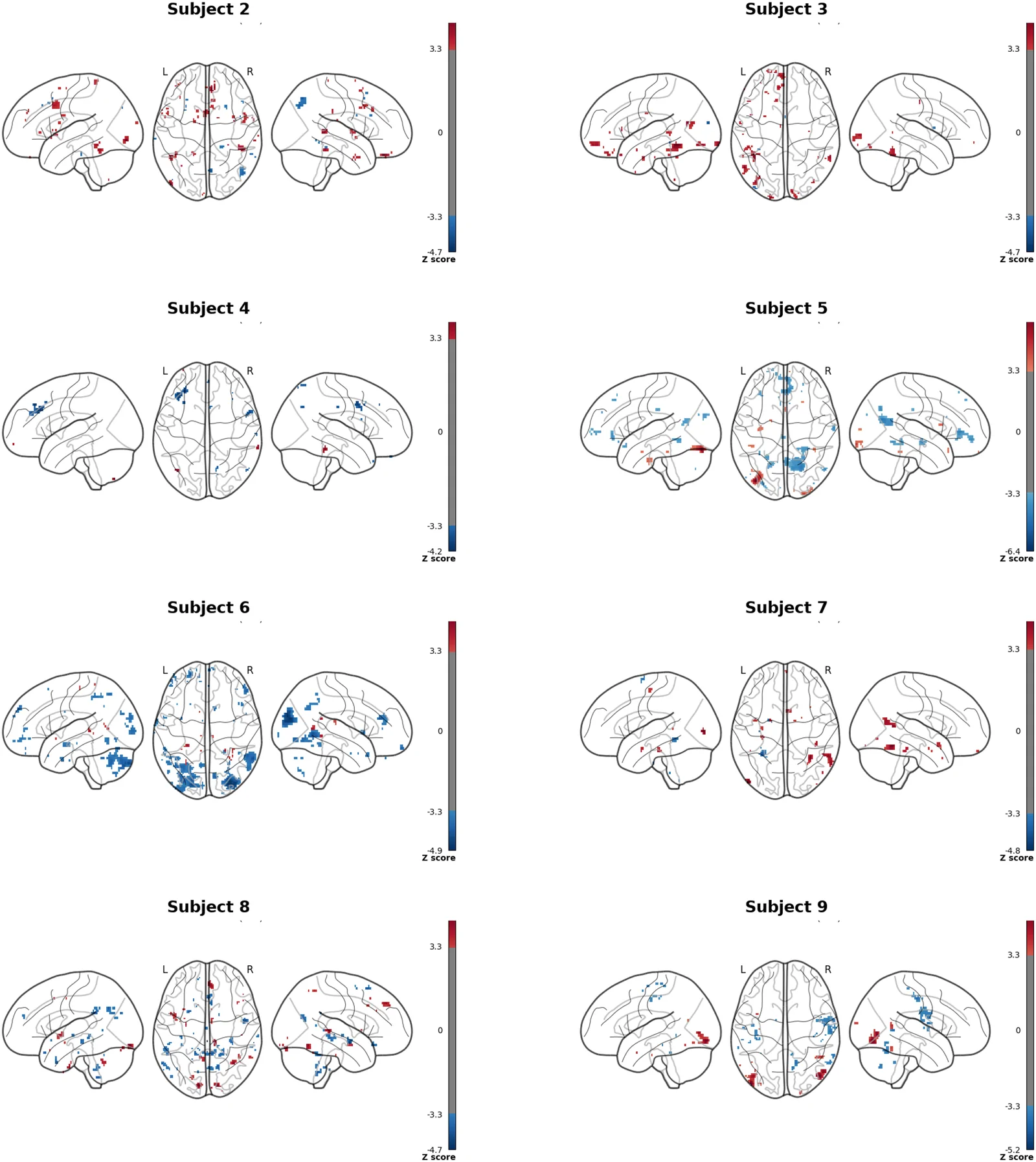
Individual subject-level activation maps for the interaction of modality and compositionality. The respective *z*-score map show the spatial distribution of significant voxels, thresholded at *p* < 0.001 uncorrected with no cluster-level correction, and a minimum peak distance of 8 mm. Positive *z*-scores (shown in red) indicate that the difference between high and low compositional complexity is significantly larger for images, whereas negative *z*-scores (shown in blue) indicate that the difference between high and low compositional complexity is significantly larger for text.

## DISCUSSION

The overall aim of this fMRI study was to characterize the neural basis of processing compositional complexity in text and images. We tested two competing hypotheses: As the main hypothesis, we proposed the existence of a shared neural basis for processing compositional complexity in text and images, reflected in shared regions in the univariate analysis or above chance MVPA decoding performance for low versus high compositional complexity. Alternatively, we proposed that distinct regions could be involved in processing compositional complexity in text and images, respectively, based on the identification of specific regions involved in either text- or image-based compositional processing but not in the cross-modal setup. Under the conceptual framework of compositional complexity employed in this study, the outcomes provided no evidence in support of either hypothesis. Neither the univariate analysis nor the MVPA yielded shared neural responses for compositional processing across modalities. Moreover, we did not find significant neural activation at the group level in regions traditionally believed to be involved in compositional processing in language such as the LIFG (Kuperberg et al., 2003; Turker et al., 2023) or ATL (Lambon Ralph et al., 2017). Across all compositionality-specific contrasts (within and across modalities, and the interaction), no consistent patterns of significant activation emerged even at the subject-specific level (see Figures 5–8).

Only for the contrast between the two input modalities, we observed clusters showing greater activation for images relative to text, or vice versa, regardless of their compositional complexity. For images, the group-level map (see Figure 2A) revealed significant activation along the well-studied ventral visual stream (Kravitz et al., 2013; Ungerleider & Haxby, 1994) including the FFA (Kanwisher et al., 1997) and PPA (Epstein & Kanwisher, 1998), among other regions. Surprisingly, for the reverse, i.e., regions showing higher activations for text relative to images, clusters of significant activation were observed in the cerebellum, anterior putamen and right caudate nucleus (see Figure 2B). The cerebellum is associated with motor control (Middleton & Strick, 1998), therefore, the activation may in part be related to eye movement (Robinson & Fuchs, 2001). Similarly, eye movement may also explain activations in the right caudate nucleus and anterior putamen (Coiner et al., 2019). Moreover, previous studies found that the caudate might be involved in language switching (Hosoda et al., 2012; Wang et al., 2007), possibly reflecting the fact that the study participants were Dutch native speakers reading English text.

A key strength of the presented study is the rigorous control of potential confounds in the data-driven stimulus selection process. By controlling for caption length and the number of nodes in the associated AMR graphs for text stimuli, and for general image density, aspect ratio, image quality, number of people and number of nodes in the associated COCO-A graphs for image stimuli, we substantially reduced the possibility that any observed differences could be attributed to low-level linguistic or perceptual variations between conditions. In particular, by matching the number of nodes in AMR graphs across highly and lowly compositionally complex text stimuli, and the number of nodes in COCO-A graphs across highly and lowly compositionally complex image stimuli, respectively (see Table 2), we explicitly isolated the depth in the hierarchical composition of concepts (Hupkes et al., 2020) rather than their mere quantity. Furthermore, the decoupling of text-image pairs within subjects (i.e., no subject viewed the matching image for a given caption and vice versa) avoided that participants imagine the corresponding image when reading a caption, and vice versa. Therefore, the null result of this study may point to the operationalization of compositional complexity (i.e., the translation of compositionality theory to quantifiable graph-based properties) as the more likely explanation for the absence of neural signatures, discussed in more detail in the following paragraph.

Specifically, the operationalization raises the fundamental question whether there are regions in the brain that encode differences in compositional complexity at all. Importantly, the depth-based operationalization employed in this study represents one possible definition of compositional complexity, and alternative accounts, such as one based on the total number of compositional operations (e.g., in multiple shallow disconnected graph components), may yield different conclusions. Moreover, the depth-based definition and contrast of compositional complexity (i.e., a depth of either 1 or 2), together with the fact that the utilized AMR and action graphs always encode at least one relational structure, did not allow for contrasting against a zero-compositionality condition within the current study design. As a result, the possibility remains that the brain does not encode differences in compositional complexity, but rather uniformly responds to the presence versus absence of the defined compositional hierarchies. Such a distinction would require a zero-compositionality baseline, similar to previous studies on meaningful two-word phrases (e.g., “red boat”) versus meaningless two-word lists (e.g., “cup, boat”, Bemis & Pylkkänen, 2011, also see Zaccarella & Friederici, 2015), which represent the presence versus absence of a relational structure. Further iterations of the proposed paradigm should explore how an additional zero-compositionality condition can be realized specifically in a multimodal experimental setting.

Beyond the absence of a zero-compositionality baseline, the resulting compositional complexity levels being either exactly (1, 1) or (2, 2) may not have been sufficiently contrastive for the extraction of robust neural response estimates as well, suggesting that the sampled range of compositional complexity may be too narrow, and that a larger range (e.g., 1 versus 5) may yield detectable effects. Additionally, the dividing line between low and high compositional complexity as operationalized in the current study may not correspond to a neuroscientifically meaningful boundary. Prior studies have established associations between human ratings of perceived (reading) complexity and syntactic properties such as parse tree frequency (Kauchak et al., 2017) or depth (Iavarone et al., 2021). However, without additional behavioral validation of the definitions employed in the current study, such as participant ratings of perceived compositional complexity (high versus low) confirming the data-driven depth labels, it remains challenging to assess the neuroscientific validity of the proposed categorical distinction between highly and lowly compositionally complex examples.

Overall, the study is subject to several limitations that warrant consideration. In particular, the aforementioned control of additional confounds substantially reduced the candidate data set (namely to 957 unique images), potentially yielding a stimulus set too small for the extraction of examples with strongly contrasting compositional complexity. Furthermore, while the COCO-A data (Ronchi & Perona, 2015) represents a more curated set of image-based scene graphs with potentially fewer quality issues compared to the commonly employed Visual Genome (Krishna et al., 2017; Milewski et al., 2020) (for more details, see Stimuli), remaining annotation quality concerns might have persisted. For instance, a COCO-A graph may potentially contain inconsistent or redundant node or edge labels, since it was generated from annotations from multiple crowd-source workers. As a result, possibly noisy graph representations may have introduced variability in the data-driven compositional complexity measures, potentially influencing the measured neural responses.

Similarly, for the text-based AMR graphs, it is challenging to estimate how accurate the automatically generated AMR graphs were for captions (rather than sentences used in the training process of AMRBart, Bai et al., 2022) in the absence of a gold standard, i.e., human-annotated AMR graphs, an aspect that may have further impacted the measured neural responses. Existing benchmarks, however, help to provide an indication of AMR parsing performance and known failure modes. AMRBart achieved a Smatch score of up to 0.854 on the AMR2.0 and AMR3.0 benchmarks (Bai et al., 2022). The Smatch score is an F1-based metric that quantifies the overlap between the concepts and relations in the automatically generated and human-annotated AMR graphs, ranging from 0 to 1. Additionally, the Granular AMR Parsing Evaluation Suite (GrAPES) (Groschwitz et al., 2023) is a more fine-grained benchmark designed to evaluate the performance of AMR graph generation models for a range of specific linguistic phenomena. While AMRBart substantially outperforms rule-based baselines on structural generalization (i.e., previously unseen compositions), it also revealed that systemic failures persist, specifically for sentences including nested complementizer phrases (i.e., phrases including multiple conjunctions such as “that”, “if”, “whether”) and coreferences in combination. Nonetheless, it is important to note that such constructions are rather unlikely given the relatively short captions included in this study.

More broadly, the experimental design decision to prioritize a larger number of stimuli per subject resulted in a trade-off regarding the included number of subjects, likely limiting the ability to detect significant effects in the conducted univariate analyses and MVPA, particularly at the group level. This challenge was further compounded by the decoupling of text-image pairs within subjects, which further increased the difficulty of the image-to-text and text-to-image MVPA decoding tasks. Next, because the lure stimuli for the behavioral recognition task were also drawn from the COCO-A data set, they were inherently constrained to semantically similar content (i.e., human visual actions in natural scenes). While the reported above-chance performances on the recognition task (see Table 3) indicate that participants paid attention, more carefully constructed lures, for instance through subtler manipulations of the original stimuli, could have provided a more informative measure of semantic processing beyond recognition memory. Additionally, it is worth noting that given the CEFR B1-C2 English proficiency range of the L2 participants, the chosen presentation rate of 3 s per text stimulus (i.e., around 250 to 375 ms per word) may have been too fast for comfortable reading for some participants, particularly during the first repetitions. Finally, considerable cognitive demand of the relatively long scanning sessions, lasting approximately 1 hr for each of the three sessions, may have introduced biases caused by participant fatigue or reduced attention. Future studies may consider a larger number of shorter scanning sessions to mitigate this limitation.

Several questions remain unanswered, given the null findings of the undertaken study. To gain additional insights into the collected data set, future studies could employ neural en-/decoding approaches using vision models, large language models or vision-language models (Oota et al., 2024) to represent and test hypotheses (Kanwisher et al., 2023) about compositional processing across modalities, in particular about possible modality-invariant representational geometries for compositional structures. Beyond the natural stimuli presented in this experiment, future experimental designs could also potentially test combinations of a controlled set of concepts in a more systematic manner to test compositional properties such as productivity (Hupkes et al., 2020) more explicitly, similar to existing compositional benchmarks for VLMs such as Winoground (Thrush et al., 2022). Winoground consists of image-caption pairs in which the same words are arranged in different compositional structures (e.g., “some plants surrounding a lightbulb” versus “a lightbulb surrounding some plants”, with accompanying images). As a result, such data could be employed to study and isolate differences in compositional structure among the same concepts. At the time of writing, Winoground-based stimuli are explored in the context of an fMRI study (van Noort et al., 2025).

Further research is also needed to find more accurate characterizations and data-driven quantifications of compositional processing. Specifically, the currently employed characterization of compositional complexity, based on graph depth rather than the number of nodes, carries a strong implicit bias toward a hierarchical notion of compositionality. At its core, this notion assumes that more complex expressions can be constructed by recursively nesting simpler expressions within one another (e.g., “The dog saw the man chase the frisbee”, dog → man → frisbee, see Introduction). However, this hierarchical assumption is increasingly challenged in recent work, suggesting that complex expressions could be instead constructed from parallel or sequential combinations of concepts (Baggio, 2021; Varaschin & Culicover, 2024). As a result, the employed graph formalism in the form of AMR and action graphs might not sufficiently capture compositional complexity. One possible alternative is to specify linear algebraic combinations of semantic or visual concepts (Efird et al., 2024; Ferrante et al., 2025). Another more fundamental aspect in the characterization concerns the distinction between studying the product of composition (i.e., the graph-based representation), the account realized in this study, compared to the process (i.e., number of operations required to build a compositional expression). Future studies targeting the latter more explicitly, for example through tasks requiring participants to actively produce compositional expressions in a multimodal setting in the scanner, may yield detectable neural signatures of compositional processing beyond the reported null results of this study. Taking the outlined methodological and theoretical considerations into account, future research efforts are needed to determine how the human brain integrates complex multimodal information, a fundamental open question at the intersection of neuroscience, artificial intelligence, and cognitive science. In the long term, such insights may inform clinical approaches to understanding and treating patients with language, vision, and semantic processing deficits.

## ACKNOWLEDGMENTS

The authors would like to thank Ronald Peeters for his feedback on the study design and the participants for their participation in the study.

## FUNDING INFORMATION

Helena Balabin, Fonds Wetenschappelijk Onderzoek (https://dx.doi.org/10.13039/501100003130), Award ID: 1154623N. Antonietta Gabriella Liuzzi, Fonds Wetenschappelijk Onderzoek (https://dx.doi.org/10.13039/501100003130), Award ID: 1247821N. Rik Vandenberghe, KU Leuven (https://dx.doi.org/10.13039/501100004040), Award ID: C14/21/109.

## AUTHOR CONTRIBUTIONS

**Helena Balabin**: Conceptualization: Lead; Data curation: Lead; Formal analysis: Lead; Investigation: Lead; Methodology: Lead; Software: Lead; Writing – original draft: Lead. **Antonietta Gabriella Liuzzi**: Methodology: Supporting; Supervision: Supporting; Writing – review & editing: Supporting. **Kevin Statz**: Data curation: Supporting; Validation: Supporting; Writing – review & editing: Supporting. **Marie-Francine Moens**: Conceptualization: Supporting; Methodology: Supporting; Supervision: Equal; Writing – review & editing: Equal. **Patrick Dupont**: Conceptualization: Supporting; Methodology: Equal; Supervision: Equal; Writing – review & editing: Equal. **Rik Vandenberghe**: Conceptualization: Lead; Funding acquisition: Lead; Investigation: Lead; Methodology: Lead; Project administration: Lead; Resources: Lead; Supervision: Lead; Writing – review & editing: Lead.

## DATA AND CODE AVAILABILITY STATEMENTS

The approved Stage 1 protocol, laboratory log, stimuli and behavioral results can be found on the Open Science Framework: https://osf.io/ta45d/overview?view_only=439003edb48c4053ab464d000894fa3e. Furthermore, the pre-processed study data supporting the reported analysis is available at Zenodo: https://zenodo.org/records/18831683 and https://zenodo.org/records/18832298. All code used for the pre-registered analyses is publicly available as well: https://github.com/lcn-kul/compositionality-study.

## Supplementary Material


