# Supplementary material for: Compositional Complexity in Text and Images
Source: Neurobiol Lang (Camb). 2026 Jul 9;7:NOL.a.271. doi: 10.1162/NOL.a.271 (PMC13506228; doi:10.1162/NOL.a.271)
Supplement: Supplementary file 1 [file nol-07-271-s001.pdf]

Supplementary Material: Compositional Complexity in Text and Images

Control for Confounding Variables: Univariate Analysis

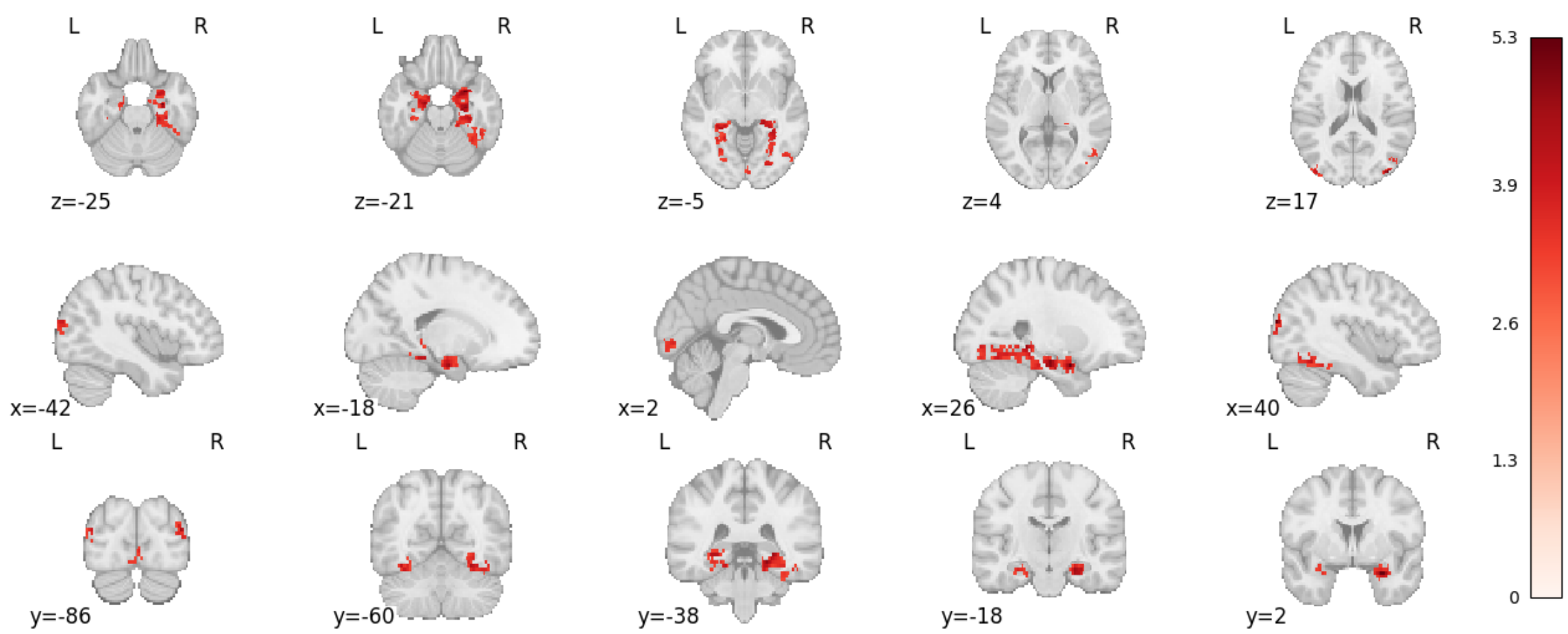

(a) *Regions showing greater activity for the one-sided contrast Image > Text.*

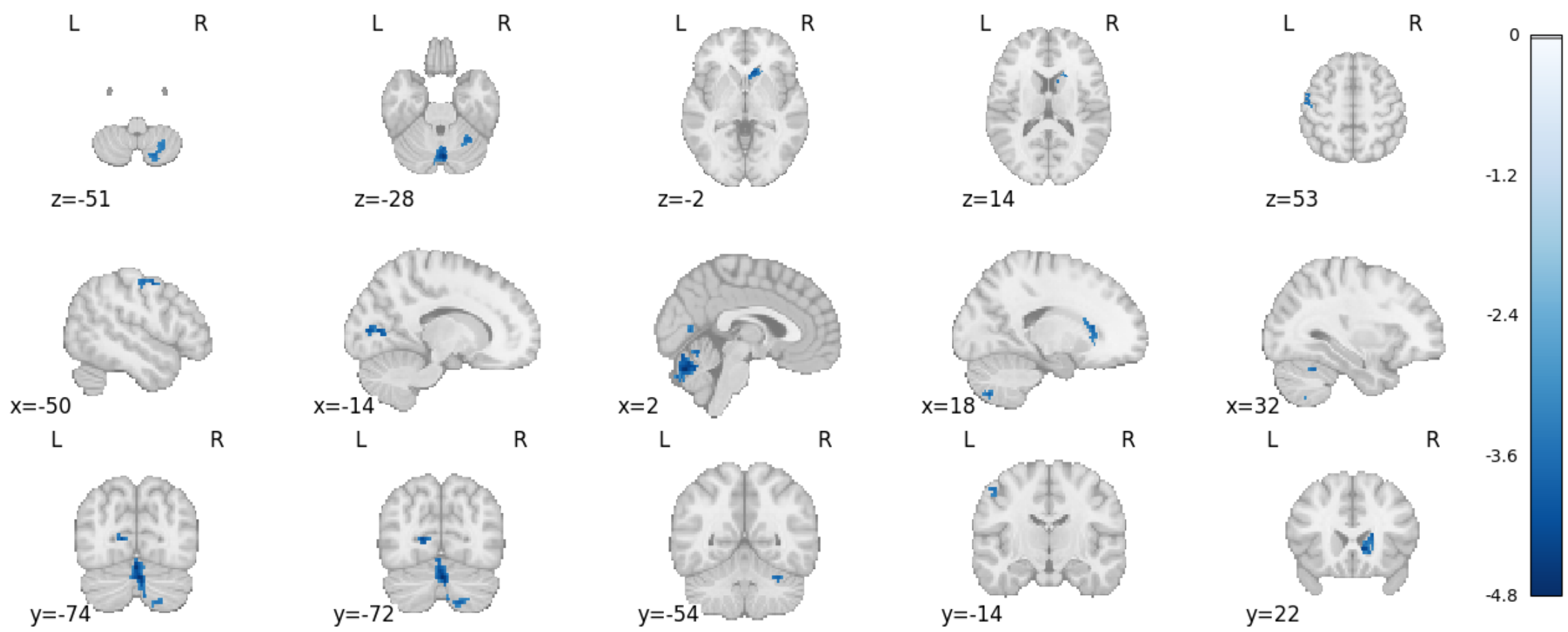

(b) *Regions showing greater activity for the one-sided contrast Text > Image.*

Figure A1

Univariate group-level main effect of modality controlling for extra confounds: caption length and the number of nodes in the associated AMR graphs for text stimuli, and general image density, aspect ratio, image quality, number of people and number of nodes in the associated COCO-A graphs for image stimuli. L = left, R = right. The statistical thresholds employed are voxel-wise $p < 0.001$ *and cluster-level family-wise error correction of* $p < 0.05$*. Results are displayed on the MNI152 template.*

Control for Confounding Variables: Multivariate Pattern Analysis

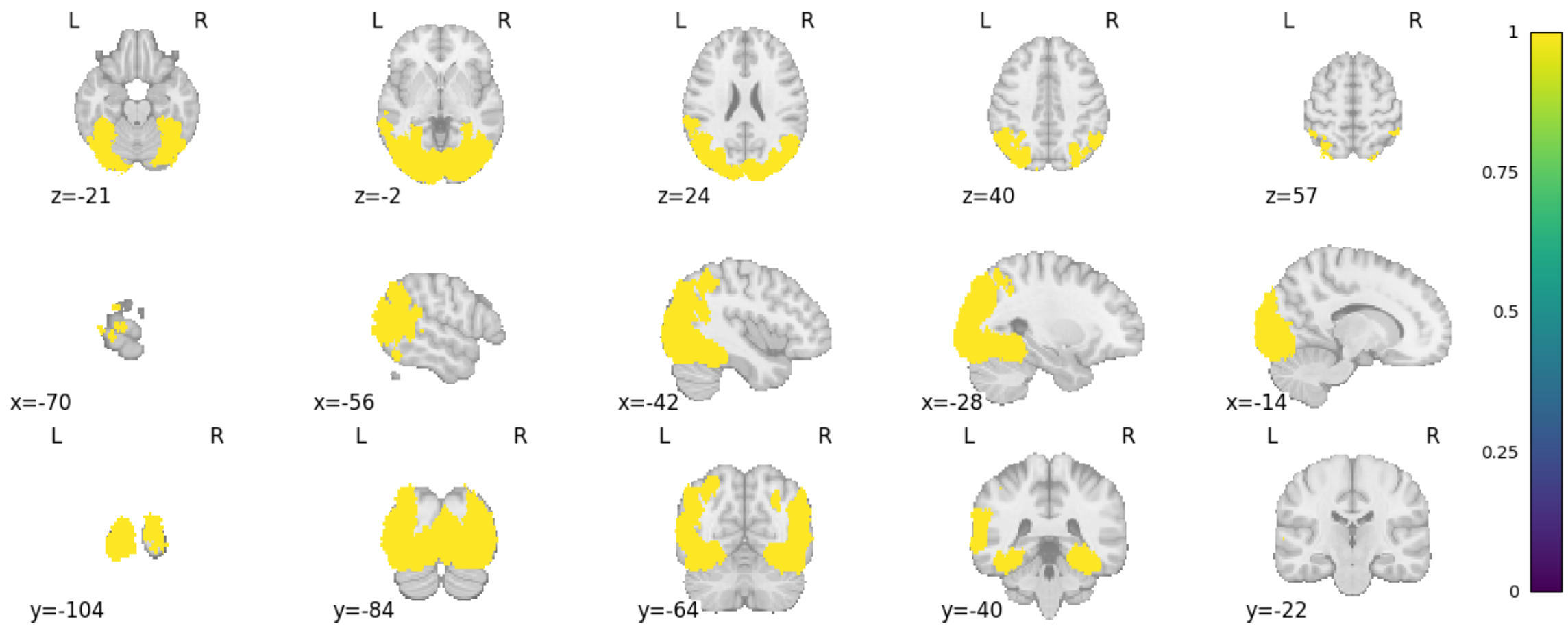

Figure A2

Group-level union of subject-level brain masks based on voxels that pass the default explained variance and test-retest variability threshold based on the GLMSingle run controlling for extra confounds: caption length and the number of nodes in the associated AMR graphs for text stimuli, and general image density, aspect ratio, image quality, number of people and number of nodes in the associated COCO-A graphs for image stimuli. L = left, R = right. Results are displayed on the MNI152 template.
